# Topical NSAIDs impact on macular oedema and visual outcome after phacoemulsification: systematic review of RCTs with network meta-analysis

**DOI:** 10.1038/s41433-024-03268-x

**Published:** 2024-08-02

**Authors:** Malaz Almasri, Abdulrahman Ismaiel, Iulia Gavris, Daniel-Corneliu Leucuta, Monica M. Gavris, Simona Delia Nicoara

**Affiliations:** 1 Department of Ophthalmology of Military Emergency Hospital “Dr. Constantin Papilian”, Cluj-Napoca, 400132 Romania; 2 Department of Ophthalmology, “Iuliu Hatieganu” University of Medicine and Pharmacy, ClujNapoca, 400394 Romania; 3 2nd Department of Internal Medicine, “Iuliu Hatieganu” University of Medicine and Pharmacy, Cluj-Napoca, 400006 Romania; 4 “Iuliu Hatieganu” University of Medicine and Pharmacy, Cluj-Napoca, Romania; 5 Department of Medical Informatics and Biostatistics, “Iuliu Hatieganu” University of Medicine and Pharmacy, Cluj-Napoca, 400349 Romania; 6 Department of Ophthalmology of County Emergency Hospital, Cluj-Napoca, 400394 Romania

**Keywords:** Retinal diseases, Lens diseases

## Abstract

The aim of this Network Meta-analysis was to compare the efficacy of the different topical Nonsteroidal anti-inflammatory drugs (NSAIDs) when added or not to topical steroids in preventing the thickening of the macula and their impact on visual acuity and intraocular pressure after phacoemulsification. Five electronic databases were searched, including PubMed, Embase, Scopus, Cochrane Library, and ClinicalTrials.gov. Our primary outcome was one-month post-surgery visual outcome. We also considered change in Foveal thickness (FT) and Intraocular pressure (IOP) at one-month post-surgery. We summarized our analyses by calculating the mean differences (MD) with associated 95% confidence intervals (CI) using restricted maximum likelihood in random effects models for continuous outcomes. The methodological quality of the studies was assessed with Cochrane Collaboration’s tool. The network meta-analysis was conducted using frequentist approach considering Nepafenac 0.1% as a reference medication. Eleven Randomized controlled trials (RCTs) including 2175 subjects were selected for quantitative analysis. At one-month post-surgery, Bromfenac had statistically significant better visual acuity compared to Nepafenac 0.1% (*p* < 0.001), regarding FT, Nepafenac 0.3% had the least increase in FT compared to Nepafenac 0.1% (*p* = 0.09), regarding IOP, Diclofenac had the lowest IOP. No significant results regarding FT and IOP. Interestingly Ketorolac had the worst results regarding BCVA and IOP, and came last but one for FT. Overall, our network meta-analysis demonstrated that Bromfenac was associated with a significant improvement in visual acuity compared to Nepafenac 0.1% at one-month following cataract surgery, while Nepafenac 0.3% was associated with the least increase in foveal thickness.

## Abstract (zh)

旨在比较局部使用不同非甾体类抗炎药物 (NSAIDs) 与合用局部类固醇在白内障超声乳化术后对黄斑区视网膜增厚以及对视力和眼压的影响。

共检索五个电子数据库, 包括 PubMed、Embase、Scopus、Cochrane Library 和 ClinicalTrials.gov。主要研究目的是评价术后一个月的视觉效果。此外还考虑了术后一个月黄斑中心凹厚度 (FT) 和眼压 (IOP) 的变化。对于连续性结果, 在随机效应模型中使用限制性最大似然法计算平均差 (MD)相关95% 置信区间 (CI)用以总结分析结果。使用Cochrane Collaboration’s tool评估研究质量。网络荟萃分析采用频率法, 0.1%奈帕芬酸作为对照。本研究共纳入11项随机对照试验 (RCTs), 共2175名受试者纳入定量分析。术后一个月, 与使用0.1%奈帕芬酸的患者相比, 使用溴芬酸的患者视力显著提高, 具有统计学意义 (p < 0.001)；与0.1%奈帕芬酸相比, 使用0.3%奈帕芬酸的患者FT增加最少 (*p* = 0.09)；使用双氯芬酸的患者IOP最低。FT与IOP之间无显著差异。局部使用酮咯酸的患者BCVA和IOP的结果最差, 而在FT的疗效中则排名最后。

综上所述, 网络荟萃分析表明, 与0.1%奈帕芬胺相比, 使用溴芬胺能明显改善白内障术后一个月的视力, 而使用0.3%奈帕芬胺与黄斑增厚的相关性最小。

## Introduction

Pseudophakic cystoid macular oedema (PCMO) is a common complication following cataract surgery, which can lead to vision loss, and there is no total consensus about its prophylaxis and treatment [1, 2].

Both topical nonsteroidal anti-inflammatory drugs (NSAIDs) and topical steroids have been used in the prevention and treatment of PCMO [3]. However, recent evidence suggests that topical NSAIDs are more efficient than topical steroids in the prevention of PCMO after phacoemulsification, especially in patients with Diabetes Mellitus [4].

The mechanism of action of these topical NSAIDs is similar, with all of them inhibiting the cyclooxygenase (COX) enzyme and reducing the production of prostaglandins, which are involved in the inflammatory response [5]. However, the different NSAIDs have varying degrees of COX-2 selectivity, duration of action, and side effect profiles, which may affect their efficacy in preventing PCMO [6]. Nepafenac 0.1%, Nepafenac 0.3%, Bromfenac, Diclofenac, and Ketorolac are commonly used topical NSAIDs for PCMO prevention [7].

There is limited evidence on the comparative efficacy of these topical NSAIDs, and head-to-head trials between all these drugs have not been conducted. Therefore, a network meta-analysis is needed to combine the evidence from existing RCTs (Randomized controlled trials). This approach provides a more complete picture of the relative efficacy of each drug, as it considers both direct and indirect comparisons. The aim of this Network Meta-analysis was to compare the efficacy of the different topical NSAIDs in combination with topical steroids or not in preventing the thickening of the macula and their impact on visual outcome after one-month of cataract surgery.

## Methods

The protocol of this systematic review was registered in INPLASY (International Platform of Registered Systematic Review and Meta-analysis Protocols); registration number (INPLASY202380078). This systematic review and network meta-analysis was written in accordance with The PRISMA Extension Statement for Reporting of Systematic Reviews Incorporating Network Meta-analyses of Health Care Interventions [8]. The Declaration of Helsinki’s principles were followed throughout all the study. Permission particular to a patient was not required.

### Eligibility criteria

Our network meta-analysis and systematic review’s inclusion criteria, in a PICOS like structure, for original papers were as follows: (1) patients undergoing cataract surgery; (2) two or more topical nonsteroidal anti-inflammatory drugs in preventing PCMO; (3) reporting baseline and one-month postoperative values of Foveal thickness (FT) and/or Best corrected visual acuity (BCVA) and/or Intraocular pressure (IOP) (4) and randomized controlled trials. Studies that met the following criteria were disregarded: (1) uncontrolled interventional studies; (2) observational studies; (3) editorials, letters, short surveys, commentaries, case reports, conference abstracts, review articles, practice guidelines, and abstracts published without a full article.

### Information sources and search strategy

Five electronic databases were searched, including PubMed, Embase, Scopus, Cochrane Library, and ClinicalTrials.gov, for studies comparing the effectiveness of different NSAIDs in the prevention of macular swelling. The search strategy included the following keywords (“Phacoemulsification”) AND ((“Nepafenac” AND “Ketorolac”) OR (“Ketorolac” AND “Diclophenac”) OR (“Diclophenac” AND “Bromfenac”) OR (“Bromfenac” AND “Nepafenac”) OR (“Nepafenac” AND “Diclofenac”) OR (“Ketorolac” AND “Bromfenac”)). The Supplementary file provides a description of the utilized search strategies for each database. Additionally, we carefully checked the papers’ references to see if there were any relevant publications missing.

### Study selection

Two researchers (M.A. and D.C.L.) independently conducted the literature search from the beginning through October 10, 2022. In the case of divergent views, a decision was made based on consensus. We didn’t apply any filters to the search strategy or place any limitations on the language, location, or time frame. We examined the complete texts of the publications that fulfilled our inclusion and exclusion criteria after doing eligibility checks on the titles and abstracts.

### Data collection process and data items

The data was extracted by one researcher (M.A.), and verified by another (D.C.L.), and any discrepancies were resolved by reading the original article. Author names, publication year, country, sample size, mean age, sex distribution, perioperative intervention for all patients, change (baseline - postoperative) in mean and standard deviation in FT and postoperative mean and standard deviation of BCVA, and IOP were among the retrieved data. BCVA that had been recorded as ETDRS letters or Snellen score were converted to Logarithm of the Minimum Angle of Resolution (logMAR) [9]. Ketorolac with concentrations of 0.4%, 0.45%, and 0.5% were assigned to the same group, Bromfenac with concentrations of 0.09% and 0.07% were assigned to the same group, Nepafenac 0.1% and Nepafenac 0.3% were assigned to different groups.

### Risk of bias within individual studies

The Cochrane Collaboration’s tool was used to assess the bias risk for randomized controlled studies [10]. Independently, each study’s risk of bias was evaluated by two writers (M.A. and D.C.L.). A discussion was conducted to reach a conclusion when there was a disagreement.

### Summary measures

The main outcome was the mean difference (MD) of BCVA. The second outcome was MD of FT and IOP. The means and standard deviations for the change in FT, and of the final values for BCVA and IOP were extracted from individual studies. In case the studies presented the standard error of the mean, we computed the standard deviation. In RCTs that did not provide the mean and standard deviation (SD) change, they were calculated using the before and after values in compliance with the guidelines in the Cochrane Handbook using the correlation coefficient from the same research or, in the event, imputed from a related study.

### Statistical analyses

The network meta-analysis was carried out with R environment for statistical computing and graphics (R Foundation for Statistical Computing, Vienna, Austria), version 4.1.2, and the netmeta R package [11, 12].

We presumed clinical variation between the trials, so we used the restricted maximum likelihood to compute the estimates of the random effects model. The mean differences were estimated along with 95% confidence intervals. The network meta-analysis was performed using a frequentist approach. For all analyses, Nepafenac 0.1% was chosen as the reference group. First, we presented the structure of comparisons with the network graphs. Next, we visualized the proportion of direct and indirect evidence used to estimate each comparison. The mean path length and minimal parallelism statistics were computed to support these results. The pooled effect from direct comparisons, as well as the pooled effect from direct and indirect comparisons were computed for all comparisons and presented in a net league table. The forest plots, having as reference the Nepafenac 0.1%, were used to present the main comparisons. The treatment effect ranking was obtained using the P-scores frequentist method. The inconsistency between direct and indirect evidence was explored with a net-splitting analysis within forest plots and net heat plot. The chi squared-based Q-test and I^2^ were used to examine between-study heterogeneity. A 0.05 level of significance was used for all analyses.

## Results

### Study selection

Search results showed 452 articles (PubMed *n* = 92 articles, EMBASE *n* = 329, Scopus *n* = 31 articles, ClinicalTrials.gov *n* = 0 articles, Cochrane Library *n* = 0 articles). A total of 79 studies were removed after being determined to be duplicated. Next, the titles and abstracts of 373 publications underwent a preliminary screening to see if they met the inclusion and exclusion criteria. The screening stage resulted in the rejection of 95 papers. We thoroughly reviewed and assessed the complete contents of the remaining 15 publications. The qualitative synthesis includes 15 of the 15 full-text papers that were available, of which 11 were included in the quantitative synthesis.

### Study characteristics

The main features of the included RCTs are outlined in Supplementary Tables S1–S4. 2175 subjects in total were included in this network meta-analysis.

### Results of network meta-analysis

**Fig. 1:**
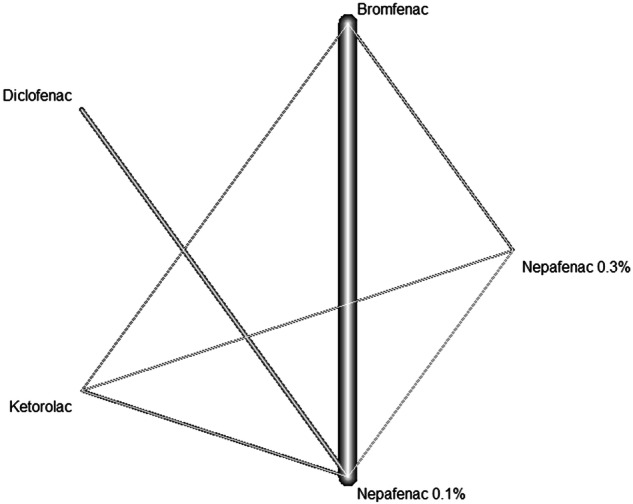
The network graph concerning BCVA.

**Fig. 2:**
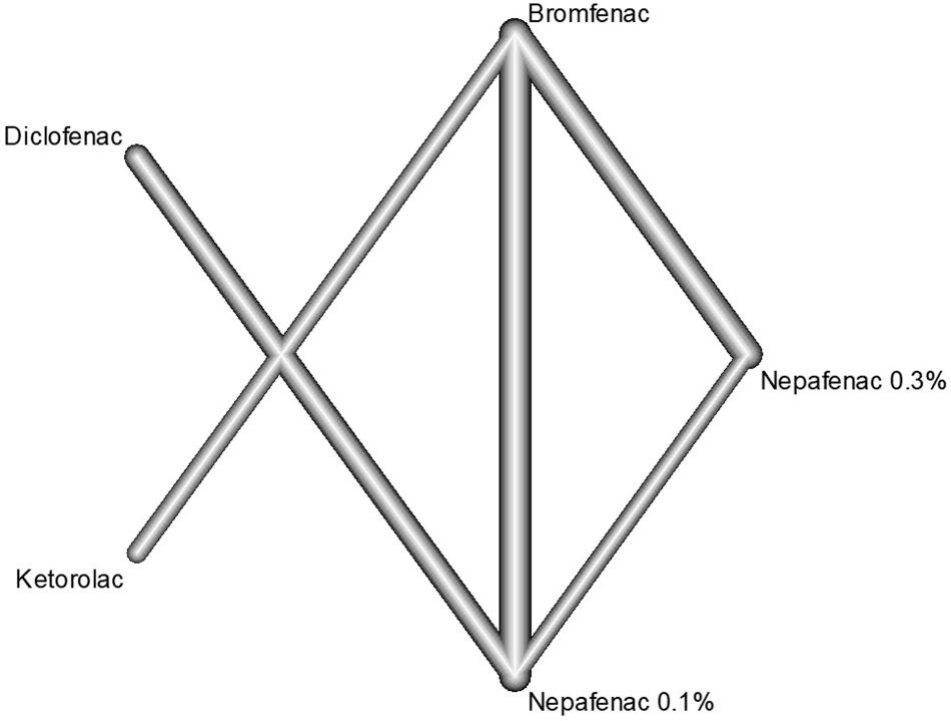
The network graph concerning foveal thickness.

**Fig. 3:**
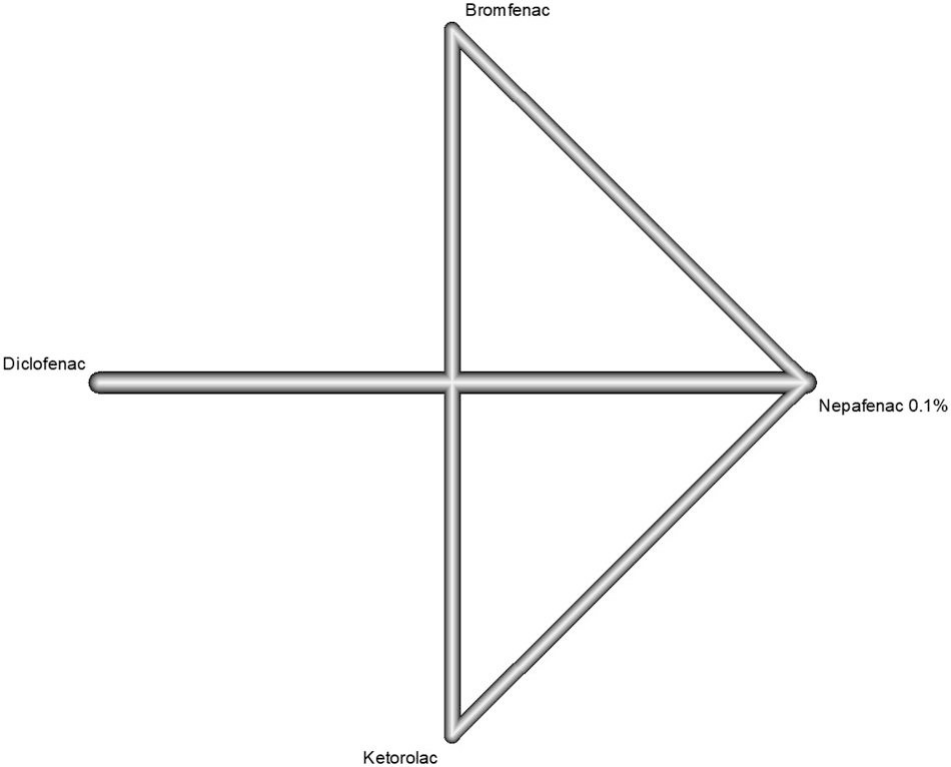
The network graph concerning intraocular pressure.

The network graph, with the overall structure of comparisons in our network, is presented in Fig. 1 for BCVA, Fig. 2 for FT, and Fig. 3 for IOP. Most of the comparisons in the selected studies were between Bromfenac and Nepafenac 0.1% regarding BCVA and FT (as observed by the degree of thickness), while regarding IOP the most frequent comparisons were between Diclofenac and Nepafenac 0.1%. Concerning the BCVA outcome, there was a reduced number of other comparisons, although the network was the most complex in terms of the variety of available head-to-head comparisons between treatments. The networks for FT and IOP were more homogenous.

**Fig. 4:**
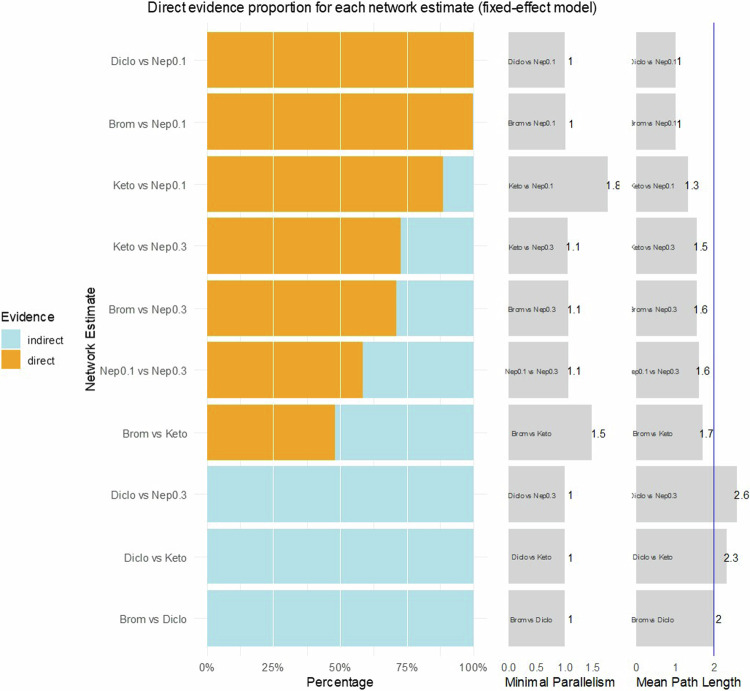
Direct evidence plot concerning BCVA. Diclo Diclofenac, Nep 0.1 Nepafenac 0.1%, Nep 0.3 Nepafenac 0.3%, Brom Bromfenac, Keto Ketorolac.

**Fig. 5:**
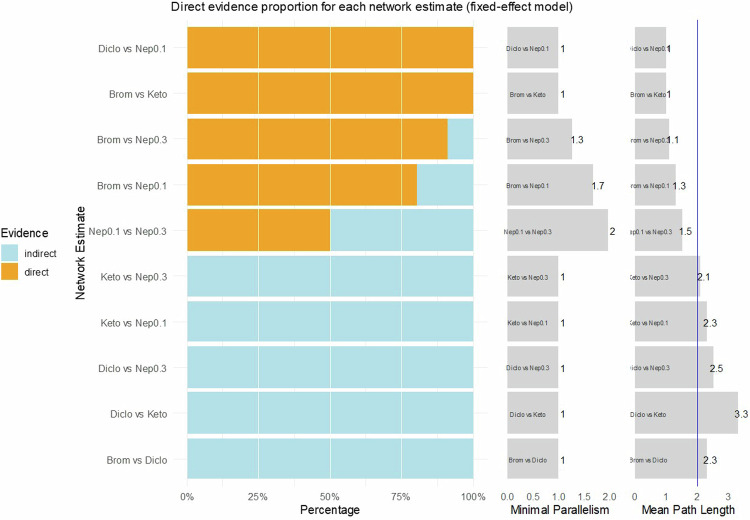
Direct evidence plot concerning foveal thickness. Diclo Diclofenac, Nep 0.1 Nepafenac 0.1%, Nep 0.3 Nepafenac 0.3%, Brom Bromfenac, Keto Ketorolac.

**Fig. 6:**
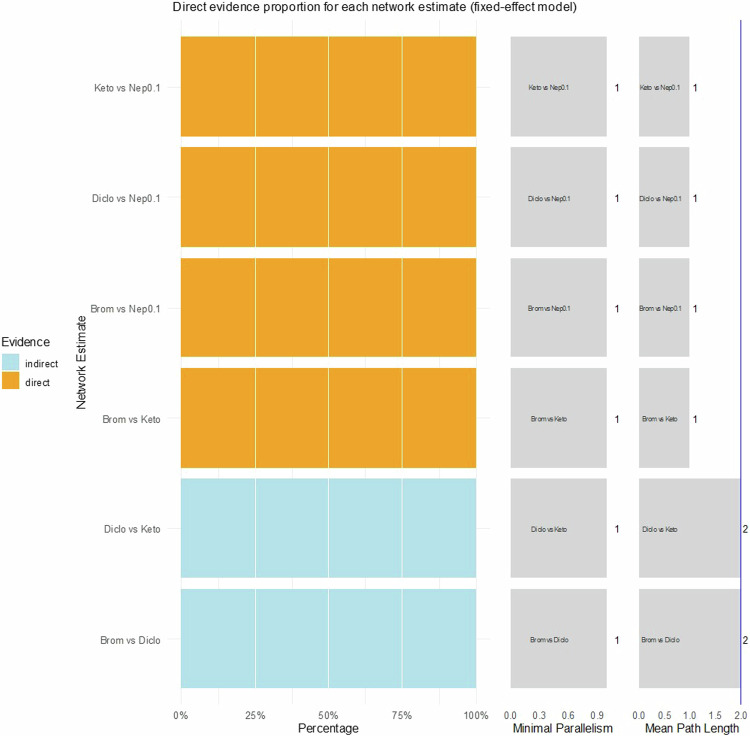
Direct evidence plot concerning intraocular pressure. Diclo Diclofenac, Nep 0.1 Nepafenac 0.1%, Brom Bromfenac, Keto Ketorolac.

The extent of direct and indirect evidence in our networks is presented in Figs. 4–6 concerning BCVA, FT, and IOP. The comparisons that should be interpreted with caution according to these analyses, are Diclofenac vs. Nepafenac 0.3% or Diclofenac vs. Ketorolac for BCVA;

Diclofenac vs. Ketorolac, Diclofenac vs. Nepafenac 0.3%, Diclofenac vs. Bromfenac, and Ketorolac vs. Nepafenac 0.1% for FT; and none for IOP, using as an indication a mean path length above 2. Regarding minimal parallelism, the largest values that support the robustness of the estimates were found for Ketorolac vs. Nepafenac 0.1% and Ketorolac vs. Bromfenac, concerning BCVA; Nepafenac 0.1% vs. Nepafenac 0.3% and Nepafenac 0.1% vs. Bromfenac, concerning FT.

All the comparisons for each outcome (BCVA, FT, IOP) are presented in the league table (Table 1).

**Table 1:** League table for all pairwise comparisons concerning BCVA, FT, and IOP.

BCVA				
Bromfenac	–	−0.01 (−0.04; 0.02)	−0.01 (−0.01; −0.01)	0.00 (−0.02; 0.03)
0.00 (−0.02; 0.02)	Diclofenac	–	−0.01 (−0.03; 0.01)	–
−0.02 (−0.05; 0.00)	−0.02 (−0.05; 0.01)	Ketorolac	0.02 (−0.01; 0.04)	0.01 (−0.02; 0.04)
−0.01 (−0.01; −0.01)*	−0.01 (−0.03; 0.01)	0.01 (−0.01; 0.04)	Nepafenac 0.1%	0.00 (−0.03; 0.03)
−0.00 (−0.03; 0.02)	−0.01 (−0.03; 0.02)	0.02 (−0.01; 0.05)	0.00 (−0.02; 0.03)	Nepafenac 0.3%
FT				
Bromfenac	–	−0.57 (−9.81; 8.67)	−0.48 (−6.91; 5.94)	6.56 (−0.06; 13.17)
0.43 (−9.51; 10.38)	Diclofenac	–	−1.93 (−9.74; 5.88)	–
−0.57 (−9.81; 8.67)	−1.00 (−14.58; 12.57)	Ketorolac	–	–
−1.50 (−7.66; 4.65)	−1.93 (−9.74; 5.88)	−0.93 (−12.04; 10.17)	Nepafenac 0.1%	5.20 (−5.03; 15.43)
5.03 (−1.22; 11.29)	4.60 (−6.27; 15.47)	5.60 (−5.56; 16.76)	6.54 (−1.02; 14.09)	Nepafenac 0.3%
IOP				
Bromfenac	–	−0.57 (−9.81; 8.67)	−0.48 (−6.91; 5.94)	6.56 (−0.06; 13.17)
0.43 (−9.51; 10.38)	Diclofenac	–	−1.93 (−9.74; 5.88)	–
−0.57 (−9.81; 8.67)	−1.00 (−14.58; 12.57)	Ketorolac	–	–
−1.50 (−7.66; 4.65)	−1.93 (−9.74; 5.88)	−0.93 (−12.04; 10.17)	Nepafenac 0.1%	5.20 (−5.03; 15.43)
5.03 (−1.22; 11.29)	4.60 (−6.27; 15.47)	5.60 (−5.56; 16.76)	6.54 (−1.02; 14.09)	Nepafenac 0.3%

*BCVA* best corrected visual acuity, *FT* foveal thickness, *IOP* intraocular pressure, The values above the diagonal represent only the pooled effect from direct comparisons, − not available, the values below the diagonal represent the pooled effect from direct and indirect comparisons.

*p-value < 0.001.

**Fig. 7:**
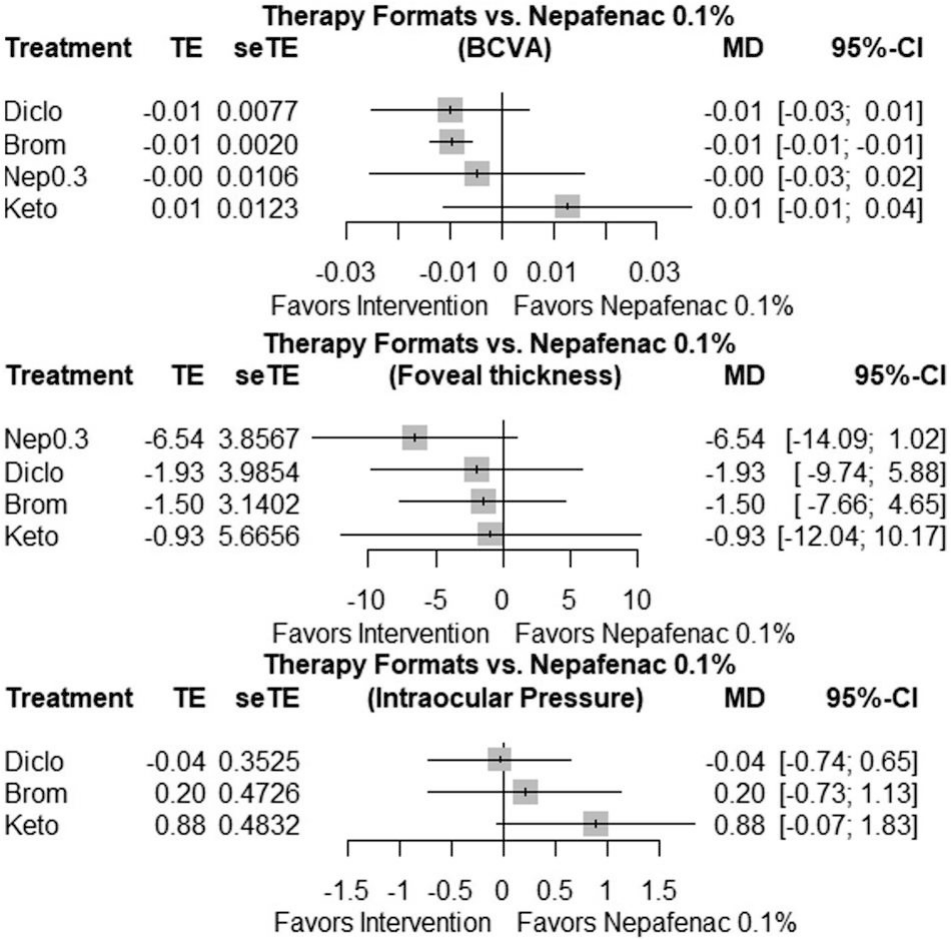
Forest plot concerning BCVA, FT, and IOP, with Nepafenac 0.1% as reference group. TE mean difference, seTE standard error of the mean difference, MD mean difference, CI confidence interval, Diclo Diclofenac, Brom Bromfenac, Keto Ketorolac.

The network meta-analysis forest plot with Nepafenac 0.1% as a reference group, observed that at one-month postoperative BCVA was statistically significant better visual acuity for Bromfenac compared to Nepafenac 0.1% (*p* < 0.001) (Fig. 7). The other comparisons didn’t show any significant result. The treatment ranking was found using the P-score ranking (Supplementary Table S5).

Regarding foveal thickness at one-month post-operatively, the network meta-analysis forest plot with Nepafenac 0.1% as a reference group, didn’t observe any significant superiority of any medication compared to Nepafenac 0.1% (Fig. 7). The treatment ranking was found using the P-score ranking showing Nepafenac 0.3% at the first place in preventing FT increase (Supplementary Table S5).

IOP network meta-analysis forest plot with Nepafenac 0,1% as reference group with the limited data available didn’t show any significant difference between the medications (Fig. 7). No comparison is available for Nepafenac 0,3%. The treatment ranking was found using the P-score ranking showing Diclofenac at the first place in lowering IOP at one-month postoperatively (Supplementary Table S5).

### Inconsistency analysis

We assessed the inconsistency between direct and indirect evidence within a net-splitting analysis.

The forest plots concerning the three outcomes are presented in Supplementary Figs. S1–S3. Results from analysing direct and indirect estimates did not indicate a major discrepancy between direct and indirect evidence. The results of the statistical tests of these comparisons were not statistically significant. Moreover, the inconsistency I^2^ values were close to 0%. Finally, we assessed the inconsistency with a net heat plot concerning BCVA (Supplementary Fig. S4), and there were no signs of inconsistency.

### The methodological quality of the included RCTs

**Fig. 8:**
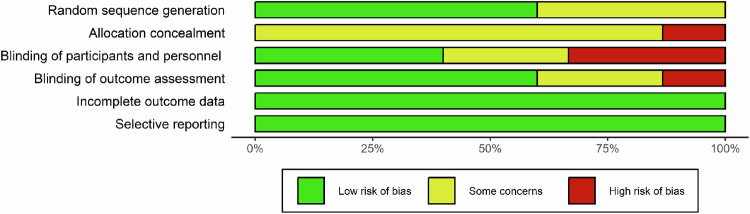
Risk of bias of the included RCTs.

In Supplementary Fig. S5 (included in the supplemental materials), the individual trials’ quality is described, while the synthesis for all trials is presented in Fig. 8. In nine (60%) of the research, the random sequence had been generated appropriately (low risk of bias); the other papers did not state how it was done (some concerns). In two (13%) studies, the allocation concealment was clearly not performed (high risk), while 13 (87%) studies did not report enough information to assess its use (some concerns). Blinding of personnel was performed in 11 (73%) of studies (low risk), unclear in two (13%) studies, and two (13%) did not use masking (high risk). Blinding of participants was performed in six (40%) of studies (low risk), unclear in four (27%) studies, and five (33%) did not use masking (high risk). Blinding outcome assessment was performed in nine (60%) of studies (low risk), unclear in four (27%) studies, and two (13%) did not use masking (high risk). All studies were assessed as having a low risk of bias concerning incomplete outcome data and selective reporting.

## Discussion

The present study aimed to compare the impact of topical Nepafenac 0.1%, Nepafenac 0.3%, Bromfenac, Diclofenac, and Ketorolac eyedrops when combined or not with steroid eyedrops on visual acuity, FT increase, IOP after one-month of phacoemulsification through a network metanalysis, considering Nepafenac 0.1% as a reference medication. Our analysis included 11 randomized controlled trials and 2175 eyes, providing a comprehensive comparison of all treatments for the three outcomes evaluated: BCVA, FT, and IOP. In our present study, Bromfenac was associated with significantly improved visual acuity at one-month post-intervention with Nepafenac 0.1% as a reference medication.

The best therapy for reducing macular inflammation has been the subject of debate in several studies. During cataract surgery, steroids are frequently prescribed because they are thought to be helpful in lowering postoperative inflammation. They also block several enzymes, such as phospholipase-A2, which are produced in high amounts after cataract surgery and thus lower arachidonic acid levels [13]. Steroids’ main downsides are increased IOP, slower wound healing, a higher risk of infection, and a difficult tapering schedule. NSAIDs have been investigated to decrease inflammation due to these disadvantages [3, 14–23]. NSAIDs are also often prescribed after cataract surgery and many RCTs have demonstrated either equal or superior efficacy of NSAIDs over topical steroids in the prevention of macular oedema [24–45]. Also a large European multicentre trial demonstrated that the combination of steroid and NSAID eyedrops reduced the risk of developing clinically significant cystoid macular oedema, although the difference was not statistically significant when compared to NSAID monotherapy [46]. According to a 2015 report by the American Academy of Ophthalmology, the greater impact of adding an NSAID to a steroid therapy may just be the result of higher dosage [23]. The safety of these drugs was not specifically evaluated in this study, but the available evidence suggests that topical NSAIDs are generally safe and well-tolerated, with a low risk of adverse events [3]. However other studies and case reports have suggested that extended use of topical NSAIDs may raise the risk of corneal melting, although it is a rare complication [47–50]. Diclofenac has been frequently linked with corneal melts [49].

Almeida et al. did a comparison between Ketorolac and Nepafenac 0.1% and suggested that the use of topical NSAIDs as a preventative measure is advised for patients who are at risk (such as those who have diabetes, retinal illness, or underwent difficult cataract surgery) in order to lessen postoperative macular oedema. And didn’t recommend using them in routine cataract surgery [24].

Tzelikis et al., Duong et al., Ramakrishnan et al., and Almeida et al. found that there is no significant difference between Ketorolac and Nepafenac 0.1% regarding visual acuity values at one-month post-surgery [24, 27, 29, 32]. Campa found that Bromfenac resulted in slightly better visual acuity at one-month post-surgery when compared to Nepafenac 0.1%, his trial had 48 eyes at each arm [26]. On the other hand, Silverstein found that Bromfenac resulted in slightly worse visual acuity at one-month when compared to Nepafenac 0.3%, his sample was about half as small [37]. Sahu compared Nepafenac 0.1% with Bromfenac and Ketorolac and found that Nepafenac 0.1% resulted in slightly better visual acuity than the other two at one-month post-surgery, followed by Bromfenac and Ketorolac. However, at 2 months, results were similar for Nepafenac 0.1% and Bromfenac and slightly worse for Ketorolac [30]. Malik performed the same comparison and found that Bromfenac resulted in better visual acuity than the other two eyedrops at one-month, followed by Ketorolac and Nepafenac 0.1% [43].

In the current study, Nepafenac 0.3% resulted in the lowest increase in foveal thickness at one-month post-surgery with Nepafenac 0.1% as a reference medication, followed by Diclofenac, Bromfenac, and Ketorolac, with minimal differences between the last three medications. All these medications were more effective than Nepafenac 0.1% in controlling post-surgery retinal inflammation. While Nepafenac 0.3% didn’t reach the significance threshold, it was close. Nepafenac 0.3% sitting on the top of the ranking in controlling foveal thickening and Nepafenac 0.1% at the bottom is confusing. The difference between the four medications Diclofenac, Bromfenac Ketorolac, and Nepafenac 0.1% were minimal. Singhal et al. found that Nepafenac 0.3% was more effective than Nepafenac 0.1%, Ketorolac, and Bromfenac in preventing FT increase [31]. On the other hand, Stock et al. found no significant difference between Nepafenac 0.3% and Ketorolac in preventing FT increase, however their study sample was too small [38]. Toyos et al. found that there is no significant difference between Nepafenac 0.3% and Bromfenac in preventing FT increase after one-month [39]. Silverstein et al. did a pilot small study and found similar results to Toyos [37].

Many RCTs resulted in minimal to no differences between the different NSAIDs regarding FT and BCVA [24, 27–29, 32, 41, 44]. Most of the RCTs included steroid medication as a control group, and compared the NSAIDs to it although adding in most of the cases a sort of steroid regimen to the NSAIDs groups. While mostly NSAIDs were superior to steroid monotherapy for most of the RCTs in preventing PCMO, comparable results between NSAIDs and steroid monotherapy were found in few RCTs [24, 32, 38]. We decided not to include steroid monotherapy as an arm in our NMA in order to evaluate the NSAIDs between themselves directly without having to rely too much on indirect evidence based on non-NSAID drugs. However, this prevented us from adding many additional RCTs with two arms, one of which being steroid monotherapy and the other being NSAID regimen, since direct comparisons between two NSAIDs or more in the same trial was far less common. A future NMA that includes steroid eyedrops in the network beside the NSAIDs could be made to include as many RCTs as possible.

Our results also show that Diclofenac had the lowest IOP after one month, followed by Nepafenac 0.1%, Bromfenac, and lastly Ketorolac with Nepafenac 0.1% as a reference medication. There weren’t sufficient data to include Nepafenac 0.3% in this analysis none of these were significant results. Ketorolac performed the worst in our analysis. Moreover, it is worth mentioning that a limited number of RCTs evaluating Ketorolac were included in this analysis due to lack of data.

One limitation was the lack of available data for some of the comparisons, which may affect the precision of the estimates. Another big limitation is the concomitant treatment given to patients in the NSAID group of each trial, in some of the trials, patients were given topical steroid during the first 3 days, or the first 2 week after the cataract surgery, some trials didn’t mention any concomitant steroid therapy. Clearly no consensus in this regard is still present. There were issues with the quality of the included studies. The most important risk of bias was related to the allocation concealment that was clearly not used in 13% of the studies, while the rest of the studies did not report any details about its use. Next, the lack of reporting of the random sequence generation was observed in 40% of studies. Nevertheless, it is less likely that the sequence generation was not appropriately generated. Blinding of participants and personnel as well as the blinding of outcome assessment was not clearly reported or not used in some of the studies. However, the subjectivity of the outcomes of interest is low.

Our study has several strengths. Firstly, network meta-analysis allows for indirect comparisons of multiple treatments, which is especially useful when there are no direct available comparisons. Secondly, we conducted a comprehensive search strategy to include as many studies as possible, enhancing the generalizability of our obtained findings. Thirdly, the use of statistical methods to evaluate the quality of evidence (minimal parallelism, consistency analysis) enhances the robustness of our findings. Lastly, the study included net-splitting analyses, as well as the fact we did not find heterogeneity between the studies further strengthens the robustness of the results.

Overall, our network meta-analysis demonstrated that Bromfenac was associated with a significant improvement in BCVA compared to Nepafenac 0.1% at one-month following cataract surgery, while Nepafenac 0.3% came in the first place in our treatment ranking with the least increase in foveal thickness, with no significant results. The differences reported for the Diclofenac, Nepafenac 0.1%, and Ketorolac were not found to be significant, but it is important to note that Ketorolac performed the poorest in terms of all our evaluated outcomes, particularly related to regulating intraocular pressure and visual outcome, although without reaching the significance threshold. Our findings could aid ophthalmologists in making informed decisions regarding the use of topical NSAIDs for the prevention of PCMO after phacoemulsification.

Further research with more head-to-head comparisons is warranted to improve the precision of our estimates.

## Supplementary information

Supplemental Material

## Data Availability

All data is available in the original articles included in the review.
