# Supplementary material for: Topical NSAIDs impact on macular oedema and visual outcome after phacoemulsification: systematic review of RCTs with network meta-analysis
Source: Eye (Lond). 2024 Aug 2;38(17):3222–30. doi: 10.1038/s41433-024-03268-x (PMC11584663; doi:10.1038/s41433-024-03268-x)
Supplement: Supplementary file 1 — Supplemental Material [file 41433_2024_3268_MOESM1_ESM.docx]

***Supplementary Tables S1 and S2*: Characteristics of included studies.**

**S1:**

| **Study** | **Study design** | **Total Subjects** | | | | | | **NSAIDs Regimen** | **Concomitant Treatment** | **Main Findings** |
| --- | --- | --- | --- | --- | --- | --- | --- | --- | --- | --- |
|  |  | **Nepafenac 0.1%** | **Nepafenac 0.3%** | **Ketorolac** | **Diclofenac** | **Bromfenac** | **Control** |  |  |  |
| **Duong et al.**  **/2007/**  **USA** | Comparative prospective double-masked clinical trial | 90 |  | 98 |  |  | NA | 3 days preoperatively: ketorolac, 1 drop 4 times a day and nepafenac, 1 drop 3 times a day.  All patients were told to continue with the regimen for 7 days after surgery. | All patients received the standard post-cataract surgery regimen of an antimicrobial agent (gatifloxacin 0.3% [Zymar], ketorolac group; moxifloxacin hydrochloride 0.5% [Vigamox], nepafenac group) 4 times a day for 7 days and a topical steroid agent (prednisolone acetate 1% [Pred Forte], ketorolac group; prednisolone acetate [Econopred], nepafenac group) 4 times a day for 7 days and tapered thereafter. The tapering regimen for both steroids was as follows: 3 times a day for 3 days, twice a day for 3 days, every day for 3 days, then discontinued completely | In conclusion, there were statistically comparable results between the Ketorolac group and the Nepafenac group for visual recovery |
| **Almeida et al.**  **/2012/**  **Canada** | Prospective placebo-controlled parallel-assignment double-masked randomized clinical trial | 54 |  | 54 |  |  | 54 | 1 drop in the operative eye 4 times a day. They began dosing 1 day before surgery and continued for 4 weeks. | All patients received gatifloxacin 0.3% drops 4 times a day starting 3 days before surgery and continued for 1 week after surgery. All patients received prednisolone 1% drops (started on day zero) 4 times a day for 1 week, 3 times a day for 1 week, 2 times a day for 1 week, and 1 time a day for 1 week. | ketorolac and nepafenac 0.1% were effective at minimizing increases in the FT and there was an overall trend toward significance, the differences were small and not significant when compared with the placebo. |
| **Tzelikis et al.**  **/2015/**  **Brasil** | Prospective placebo-controlled randomized study | 41 |  | 45 |  |  | 40 | 1 drop of ketorolac 4 times a day and 1 drop of nepafenac 0.1% 3 times a day. They began dosing 2 days before surgery and continued for 4 weeks. | All patients received moxifloxacin 0.5% drops 4 times a day starting 2 days before surgery and continued for 7 days after surgery. All patients received on day 0 prednisolone 1% drops 4 times a day for 1 week, 3 times a day for 1 week, 2 times a day for 1 week and 1 time a day for 1 week. | Placebo, Ketorolac, and Nepafenac groups all had statistically identical visual recovery,and FT. |
| **Jung et al.**  **/2015/**  **Korea** | Single-center, randomized study |  |  | 32 |  | 28 | 31 | Group 1: 1 drop of bromfenac twice daily beginning 3 days before surgery and 2 drops at 20-minute intervals at 2 hours before surgery. They continued using bromfenac twice daily with other postoperative eye drops for 4 weeks. Group 2: 1 drop of ketorolac twice-daily beginning 1 day prior to cataract surgery and 2 drops at 20-minute intervals at 2 hours before surgery, with continued application twice-daily throughout the first 2 postoperative weeks. | Postoperatively, all patients in all three groups were prescribed topical gatifloxacin 0.3% and prednisolone acetate 1% eye drops that were to be applied 4 times daily for 4 weeks. | In terms of reducing controlling macular thickness following cataract surgery, the combined treatment of a topical steroid and Bromfenac or Ketorolac is better than steroid alone. No difference were found between Bromfenac and Ketorolac (P=0.905) |
| **Sahu et al.**  **/2015/**  **India** | Single-center prospective randomized study | 31 |  | 33 |  | 30 | 26 | 2 times a day (bromfenac 0.09%) or 3 times a day (ketorolac 0.4% and nepafenac 0.1%) 1 day before surgery. On the day of surgery: 1 drop of the NSAID 3 times at 30-minute intervals. The topical NSAID was started preoperatively and continued in the same dosages in the respective groups for 6 weeks. | All patients received topical moxifloxacin 0.5% 6 times a day. The topical antibiotic (moxifloxacin 0.5%) and steroid (prednisolone acetate 1.0%) drops were administered in the same daily dosages and tapered post- operatively. The tapering regimen for the topical steroid was as follows: 4 times a day for 7 days, 3 times a day for 3 days, twice a day for 3 days, once every day for 3 days, and then discontinued. | Ketorolac, Bromfenac, and Nepafenac 0.1% reduced postoperative inflammation. No difference was found between the 4 groups regarding BCVA at 4 week post-operatively |
| **Ramakrishnan et al.**  **/2015/**  **India** | Prospective, randomized, parallel-assignment efficacy trial | 100 |  | 100 |  |  | NA | 0.1% nepafenac group: 3 times a day at 1 day preoperatively and 30 days postoperatively. Ketorolac group: 4 times a day at 1 day preoperatively and 30 days postoperatively. On the day of the surgery, all patients received the NSAID eye drop of their allocated group every 15 minutes for 1 hour before surgery. | All patients received 0.3% ofloxacin + 1% prednisolone acetate combination eye drops in tapering doses for 1 month postoperatively and 0.3% ofloxacin eye drops 4 times a day at 1 day preoperatively and for 1 week postoperatively. | Nepafenac and Ketorolac had similar effects on postoperative macular thickening following cataract surgery. |
| **Malik et al.**  **/2016/**  **India** | Prospective randomized study | 50 |  | 50 |  | 50 | 50 | Nepafenac 0.1% group: 1 drop 3 times daily for 1 month; Bromfenac group: 1 drop twice daily for 1 month; Ketorolac: 1 drop 4 times daily for 1 month. | NA | Controlling ocular inflammation following cataract surgery is possible with all four medications. Prednisolone 1% alone works best to manage intraocular inflammation, whereas NSAIDs work better to control ocular discomfort and hyperemia in the early postoperative phases. |
| **Palacio et al.**  **/2016/**  **Mexico** | Parallel, randomized, double-blind, active-controlled, multicenter clinical trial | 70 |  |  |  | 69 | NA | For both NSAIDs: 5 drops into each patient’s eye in the hour before surgery. After surgery: 1 drop 3 times daily for 30 days. | NA | In comparison to Nepafenac 0.1%, Bromfenac has shown comparable clinical effectiveness to lessen the increase of FT and in IOP results following phacoemulsification. |
| **Campa et al.**  **/2018/** | Prospective, randomized, open-label study | 48 |  |  |  | 48 | 48 | Nepafenac 0.1% group: 2 times a day and 3 days before surgery and nepafenac 3 times a day for 4 weeks after surgery; Bromfenac group: 2 times a day for 3 days before surgery; bromfenac 2 times a day for 15 days | All patients were treated preoperatively with azithromycin dihydrate (15mg/g) eyedrops 2 times a day for 3 days and a combined solution of dexamethasone and netilmicin 4 times a day for 4 weeks after surgery. | In comparison to steroid monotherapy, co-administration of Nepafenac or Bromfenac plus steroids in individuals is related with a lower FT increase. However, no difference between the 2 NSAIDs regarding FT and VA. |
| **Stock et al. /2018/**  **Brazil** | Double-blind, randomized, comparative, quantitative, prospective study |  | 21 | 32 |  |  | 24 | The eye drops were used preoperatively (2 days and 5 minutes before surgery) and postoperatively for 45 days. Nepafenac was applied 1 time per day, and ketorolac was applied 3 times per day. | NA | No significant changes in FT variation between patients who took prophylactic eye drops of Nepafenac, Ketorolac Tromethamine, or Propylene glycol. None of the patients had PME. |
| **Ylinen et al.**  **/2018/**  **Finland** | Randomized, double-blind, prospective single-centre study | 48 |  |  | 48 |  | NA | Both anti- inflammatory eye drops were used three times a day for 21 days post-operatively. | NA | No differences in clinical effectiveness across the NSAIDs were discovered. |
| **Toyos et al.**  **/2019/**  **USA** | Prospective, single site, randomized,  single-masked, parallel-group pilot study |  | 24 |  |  | 25 | NA | 1 drop daily of the assigned medication beginning 3 days before surgery, one dose on the day of surgery, then 4 times a day for 21 days after surgery. | All patients received Besifloxacin 0.6% 2 times daily prior to surgery. Intraoperatively, subjects were administered 1 drop each of prednisolone acetate 1% and moxifloxacin 0.5%, and then given Besifloxacin 0.6%, 2 times a day for 10 days post-operatively | When dosed according to the same regimens, both Bromfenac and Nepafenac 0.3% exhibited favorable and comparable clinical results in terms of visual acuity following cataract surgery. Small and comparable increases in mean macular volume and mean retinal thickness were seen between treatments. |
| **Silverstein et al.**  **/2019/**  **USA** | Single-center, randomized, investigator-masked, parallel group, active-comparator controlled pilot study |  | 25 |  |  | 24 | NA | Both medications: 1 drop once daily at the same time each day, beginning 1 day prior to surgery (day 1) and continuing on the day of surgery (day 0) and for about 14 days after surgery. On the day of surgery, 1 drop of the assigned study drug into each patient’s study eye 1 h prior to surgery. | All patients received besifloxacin 0.6% suspension 3 times daily for 2 days prior to surgery, every 2 h while awake on the day of surgery, and 3 times a day each day after surgery until the bottle was finished. | When examined using comparable dose regimens, once-daily Bromfenac had equivalent effects to once-daily Nepafenac 0.3% in terms of post-surgical inflammation, VA, and retinal thickness, and may even have a quicker beginning of anti-inflammatory activity. |
| **Cagini et al.**  **/2020/**  **Italy** | Single-centre randomized trial | 32 |  |  | 32 |  | NA | Dicofenac: 4 times daily for 4 weeks. Nepafenac 0.1%: 4 times daily for 4 weeks | After the discharge, all patients started treatment with ofloxacin 0.5% eye drops 4 times daily for the first week, tobramycin 0.3% and dexamethasone 0.1% eye drops (Tobradex) 4 times daily for 2 weeks. | No significant difference between the groups regarding BCVA, FT and IOP |
| **Singhal et al.**  **/2022/**  **India** | Prospective comparative study | 96 | 96 | 94 |  | 93 | 91 | Bromfenac: twice daily for 6 weeks; Ketorolac: twice daily for 6 weeks; nepafenac 0.1% thrice daily for 6 weeks; nepafenac 0.3% once daily for 6 weeks. | All patients received topical therapy including moxifloxacin hydrochloride 0.5% 4 times a day and carboxymethyl cellulose 1% four times a day. | Nepafenac 0.3% was statistically more efficient in reducing the FT increase after phacoemulsification when compared to Prednisolone group, the other NSAIDs group had comparable results. No difference between the medications regarding VA and intraocular pressure |

PME, Pseudophakic Macular Edema; VA, Visual Acuity; FT, Foveal Thickness; TMV, Total macular thickness; NSAIDs, Non-steroidal anti-inflammatory drugs.

All the medications mentioned are topical ophthalmic suspensions.

***S2:***

| **Study** | **Study design** | **Mean age (years)** **± Standard Deviation:** | | | | | | **Sex** | |
| --- | --- | --- | --- | --- | --- | --- | --- | --- | --- |
|  |  | **Nepafenac 0.1%** | **Nepafenac 0.3%** | **Ketorolac** | **Diclofenac** | **Bromfenac** | **Control** | **Male** | **Female** |
| **Duong et al.**  **/2007/**  **USA** | Comparative prospective double-masked clinical trial | 69.47 ± 10.67 |  | 68.92 ± 12.18 |  |  |  | 80 | 98 |
| **Almeida et al.**  **/2012/**  **Canada** | Prospective placebo-controlled parallel-assignment double-masked randomized clinical trial | NA | NA |  |  |  |  | NA | NA |
| **Tzelikis et al.**  **/2015/**  **Brasil** | Prospective placebo-controlled randomized study | NA | NA |  |  |  |  | 56 | 70 |
| **Jung et al.**  **/2015/**  **Korea** | Single-center, randomized study |  |  | 67.5 ± 7.0 |  | 66.9 ± 11.1 | 66.8 ± 8.1 | 41 | 50 |
| **Sahu et al.**  **/2015/**  **India** | Single-center prospective randomized study | 60.42 ± 10.72 |  | 63.48 ± 9.60 |  | 59.63 ± 8.96 | 60.77 ± 9.65 | 67 | 53 |
| **Ramakrishnan et al.**  **/2015/**  **India** | Prospective, randomized, parallel-assignment efficacy trial | 58 ± 8.5 |  | 59.3 ±  8.7 |  |  |  | NA | NA |
| **Malik et al.**  **/2016/**  **India** | Prospective randomized study | 59.42 ± 8.56 |  | 58.30 ± 7.89 |  | 62.60±8.64 | 59.54 ±9.49 | 76 | 124 |
| **Palacio et al.**  **/2016/**  **Mexico** | Parallel, randomized, double-blind, active-controlled, multicenter clinical trial | 68.0 ± 9.5 |  |  |  | 67.4 ± 11.1 |  | 49 | 80 |
| **Campa et al.**  **/2018/** | Prospective, randomized, open-label study | 78.21 ± 7.87 |  |  |  | 77 ± 5.93 | 78.75 ± 7.96 | 64 | 80 |
| **Stock et al. /2018/**  **Brazil** | Double-blind, randomized, comparative, quantitative, prospective study |  | NA | NA |  |  |  | NA | NA |
| **Ylinen et al.**  **/2018/**  **Finland** | Randomized, double-blind, prospective single-centre study | 75.6 ± 1.0 |  |  | 76.4 ± 0.9 |  |  | 37 | 59 |
| **Toyos et al.**  **/2019/**  **USA** | Prospective, single site, randomized,  single-masked, parallel-group pilot study |  | 66.9 ± 9.75 |  |  | 68.3 ± 8 |  | 15 | 34 |
| **Silverstein et al.**  **/2019/**  **USA** | Single-center, randomized, investigator-masked, parallel group, active-comparator controlled pilot study |  | 65.8 ± 9.82 |  |  | 67.2 ± 8.43 |  | 19 | 30 |
| **Cagini et al.**  **/2020/**  **Italy** | Single-centre randomized trial | 77.9 ± 5.6 |  |  | 76.2 ± 6.6 |  |  | 31 | 33 |
| **Singhal et al.**  **/2022/**  **India** | Prospective comparative study | 65.9 ± 8.5 | 64 ± 6.6 | 62.9 ± 11.3 |  | 62.6 ± 13.1 | 65.4 ± 9.0 | 58 | 38 |

***Supplementary Tables S3 and S4 Study outcomes***

***S3:***

| **Best corrected visual acuity** | | | | | | | | | | | | | | | | |
| --- | --- | --- | --- | --- | --- | --- | --- | --- | --- | --- | --- | --- | --- | --- | --- | --- |
| **Study** | **Nepafenac 0.1%** | | | **Nepafenac 0.3%** | | | **Bromfenac** | | | **Diclofenac** | | | **Ketorolac** | | | **Evaluation period** |
|  | **N** | **M post-surgery** | **SD post-surgery** | **N** | **M post-surgery** | **SD post-surgery** | **N** | **M post-surgery** | **SD post-surgery** | **N** | **M post-surgery** | **SD post-surgery** | **N** | **M post-surgery** | **SD post-surgery** |  |
| **Sahu** | 31 | 0.07 | 0.12 | - | - | - | 30 | 0.09 | 0.1 | - | - | - | 33 | 0.1 | 0.12 | 4 weeks |
| **Tzelikis** | 41 | 0.04 | 0.1 | - | - | - |  |  |  | - | - | - | 45 | 0.06 | 0.12 | 4 weeks |
| **Campa** | 48 | 0.05 | 0.01 | - | - | - | 48 | 0.04 | 0.01 | - | - | - | - | - | - | 5 weeks |
| **Ylinen** | 45 | 0.08 | 0.2 | - | - | - | - | - | - | 42 | 0.07 | 0.13 | - | - | - | 4 weeks |
| **Toyos** | - | - | - | 24 | -0.02 | 0.12 | 25 | 0.01 | 0.3949 | - | - | - | - | - | - | 6 weeks |
| **Silverstein** | - | - | - | 9 | 0.04 | 0.096 | 11 | 0.05 | 0.115 | - | - | - | - | - | - | 6 weeks |
| **Cagini** | 32 | 0.02 | 0.04 | - | - | - | - | - | - | 32 | 0.01 | 0.02 | - | - | - | 4 weeks |
| **Singhal** | 96 | 0.07 | 0.11 | 96 | 0.07 | 0.08 | 93 | 0.07 | 0.1 | - | - | - | 94 | 0.08 | 0.14 | 6 weeks |
| **Foveal thickness** | | | | | | | | | | | | | | | | |
| **Study** | **Nepafenac 0.1%** | | | **Nepafenac 0.3%** | | | **Bromfenac** | | | **Diclofenac** | | | **Ketorolac** | | | **Evaluation period** |
|  | **N** | **M change** | **SD change** | **N** | **M change** | **SD change** | **N** | **M change** | **SD change** | **N** | **M change** | **SD change** | **N** | **M change** | **SD change** |  |
| **Palacio** | 70 | 13.2 | 18.18 | - | - | - | 69 | 4.8 | 21.51 | - | - | - | - | - | - | 4 weeks |
| **Jung** | - | - | - | - | - | - | 28 | 4.3 | 4.25 | - | - | - | 32 | 4.87 | 6.03 | 4 weeks |
| **Campa** | 48 | 7.35 | 12.81 | - | - | - | 48 | 6 | 16.66 | - | - | - | - | - | - | 5 weeks |
| **Ylinen** | 45 | 4.7 | 21.46 | - | - | - | - | - | - | 42 | 2.6 | 7.77 | - | - | - | 4 weeks |
| **Toyos** | - | - | - | 24 | 10.6 | 3.27 | 25 | 14.7 | 5.67 | - | - | - | - | - | - | 6 weeks |
| **Silverstein** | - | - | - | 9 | 17.3 | 16.3 | 11 | 11 | 20.9 | - | - | - | - | - | - | 6 weeks |
| **Cagini** | 32 | 3.62 | 13.33 | - | - | - | - | - | - | 32 | 1.85 | 13.13 | - | - | - | 4 weeks |
| **Singhal** | 96 | 12 | 22.7 | 96 | 6.8 | 11.6 | 93 | 21.4 | 30.2 | - | - | - | - | - | - | 6 weeks |
| **Intraocular pressure** | | | | | | | | | | | | | | | | |
| **Study** | **Nepafenac 0.1%** | | | **Nepafenac 0.3%** | | | **Bromfenac** | | | **Diclofenac** | | | **Ketorolac** | | | **Evaluation period** |
|  | **N** | **M post-surgery** | **SD post-surgery** | **N** | **M post-surgery** | **SD post-surgery** | **N** | **M post-surgery** | **SD post-surgery** | **N** | **M post-surgery** | **SD post-surgery** | **N** | **M post-surgery** | **SD post-surgery** |  |
| **Malik** | 50 | 15.28 | 2.48 | - | - | - | 50 | 15.48 | 2.24 | - | - | - | 50 | 16.16 | 2.35 | 4 weeks |
| **Ylinen** | 45 | 10.5 | 2.68 | - | - | - | - | - | - | 42 | 10.7 | 2.59 | - | - | - | 4 weeks |
| **Cagini** | 32 | 13.6 | 1.7 | - | - | - | - | - | - | 32 | 13.4 | 1.9 | - | - | - | 4 weeks |

N, number of subjects; M, mean; SD, standard deviation.

***S4:***

| **BCVA: Best Corrected Visual Acuity** | | | | | | |
| --- | --- | --- | --- | --- | --- | --- |
| **TE** | **seTE** | **treat1.long** | **treat2.long** | **treat1** | **treat2** | **studlab** |
| -0.02 | 0.028246 | Nepafenac 0.1% | Bromfenac | Nep0.1 | Brom | **Sahu** |
| -0.03 | 0.030015 | Nepafenac 0.1% | Ketorolac | Nep0.1 | Keto | **Sahu** |
| -0.01 | 0.333987 | Bromfenac | Ketorolac | Brom | Keto | **Sahu** |
| -0.02 | 0.023747 | Nepafenac 0.1% | Ketorolac | Nep0.1 | Keto | **Tzelikis** |
| 0.01 | 0.002041 | Nepafenac 0.1% | Bromfenac | Nep0.1 | Brom | **Campa** |
| 0.01 | 0.035934 | Nepafenac 0.1% | Diclofenac | Nep0.1 | Diclo | **Ylinen** |
| -0.03 | 0.08331 | Nepafenac 0.3% | Bromfenac | Nep0.3 | Brom | **Toyos** |
| -0.01 | 0.047183 | Nepafenac 0.3% | Bromfenac | Nep0.3 | Brom | **Silverstein** |
| 0.01 | 0.007906 | Nepafenac 0.1% | Diclofenac | Nep0.1 | Diclo | **Cagini** |
| 0 | 0.015283 | Nepafenac 0.1% | Bromfenac | Nep0.1 | Brom | **Singhal** |
| -0.07 | 0.013198 | Nepafenac 0.3% | Bromfenac | Nep0.3 | Brom | **Singhal** |
| 0.07 | 0.013882 | Nepafenac 0.1% | Nepafenac 0.3% | Nep0.1 | Nep0.3 | **Singhal** |
| -0.01 | 0.018291 | Nepafenac 0.1% | Ketorolac | Nep0.1 | Keto | **Singhal** |
| -0.01 | 0.016588 | Nepafenac 0.3% | Ketorolac | Nep0.3 | Keto | **Singhal** |
| -0.01 | 0.017777 | Bromfenac | Ketorolac | Brom | Keto | **Singhal** |

| **FT: Foveal Thickness** | | | | | | |
| --- | --- | --- | --- | --- | --- | --- |
| **TE** | **seTE** | **treat1.long** | **treat2.long** | **treat1** | **treat2** | **studlab** |
| 8.4 | 3.3813 | Nepafenac 0.1% | Bromfenac | Nep | Brom | **Palacio** |
| -0.57 | 1.334679 | Bromfenac | Ketorolac | Brom | Keto | **Jung** |
| 1.35 | 3.034837 | Nepafenac 0.1% | Bromfenac | Nep | Brom | **Campa** |
| 2.1 | 3.417601 | Nepafenac 0.1% | Diclofenac | Nep | Diclo | **Ylinen** |
| -4.1 | 1.315862 | Nepafenac 0.3% | Bromfenac | Nep0.3 | Brom | **Toyos** |
| 6.3 | 8.320523 | Nepafenac 0.3% | Bromfenac | Nep0.3 | Brom | **Silverstein** |
| 1.77 | 3.308686 | Nepafenac 0.1% | Diclofenac | Nep | Diclo | **Cagini** |
| -9.4 | 3.895444 | Nepafenac 0.1% | Bromfenac | Nep | Brom | **Singhal** |
| -14.6 | 3.347917 | Nepafenac 0.3% | Bromfenac | Nep0.3 | Brom | **Singhal** |

| **IOP: Intraocular Pressure** | | | | | | |
| --- | --- | --- | --- | --- | --- | --- |
| **TE** | **seTE** | **treat1.long** | **treat2.long** | **treat1** | **treat2** | **studlab** |
| -0.2 | 0.47261 | Nepafenac 0.1% | Bromfenac | Nep0.1 | Brom | **Malik** |
| -0.88 | 0.483175 | Nepafenac 0.1% | Ketorolac | Nep0.1 | Keto | **Malik** |
| -0.68 | 0.459132 | Bromfenac | Ketorolac | Brom | Keto | **Malik** |
| -0.2 | 0.565685 | Nepafenac 0.1% | Diclofenac | Nep0.1 | Diclo | **Ylinen** |
| 0.2 | 0.450694 | Nepafenac 0.1% | Diclofenac | Nep0.1 | Diclo | **Cagini** |

TE, treatment effect; seTE, standard error of the effect;

**Search strategy 03.10.2022**

**Total: 452**

**Duplicates: 79**

**Total after removing duplicates: 373**

**PubMed – 92 articles**

(("Phacoemulsification"[Mesh]) OR ("Phacoemulsification"[All Fields])) AND (("Nepafenac") AND ("Ketorolac")) OR (("ketorolac") AND ("diclophenac")) OR (("diclophenac") AND ("Bromfenac")) OR (("bromfenac") AND ("nepafenac")) OR (("Nepafenac") AND ("diclofenac")) OR (("ketorolac") AND ("bromfenac"))

**Scopus – 31 articles**

(("Phacoemulsification"[Mesh]) OR ("Phacoemulsification"[All Fields])) AND (("Nepafenac") AND ("Ketorolac")) OR (("ketorolac") AND ("diclophenac")) OR (("diclophenac") AND ("Bromfenac")) OR (("bromfenac") AND ("nepafenac")) OR (("Nepafenac") AND ("diclofenac")) OR (("ketorolac") AND ("bromfenac"))

**Embase – 329 articles**

('Phacoemulsification'/exp OR 'Phacoemulsification') AND ('Nepafenac' AND 'Ketorolac') OR ('ketorolac' AND 'diclophenac') OR ('diclophenac' AND 'Bromfenac') OR ('bromfenac' AND 'nepafenac') OR ('Nepafenac' AND 'diclofenac') OR ('ketorolac' AND 'bromfenac')

**Cochrane Library – 0 article**

**Clinicaltrials.gov – 0 article**

**Supplementary Table S5.** **P-score treatment rankings for foveal thickness (FT), best corrected visual acuity (BCVA), intraocular pressure (IOP).**

|  | **FT** | | **BCVA** | | **IOP** | |
| --- | --- | --- | --- | --- | --- | --- |
| **Rank** | **Drug** | **P-score** | **Drug** | **P-score** | **Drug** | **P-score** |
| **1** | Nep0.3 | 0.8829 | Brom | 0.7824 | Diclo | 0.7168 |
| **2** | Diclo | 0.4952 | Diclo | 0.7524 | Nep0.1 | 0.6931 |
| **3** | Brom | 0.4388 | Nep0.3 | 0.5593 | Brom | 0.5353 |
| **4** | Keto | 0.4055 | Nep0.1 | 0.3177 | Keto | 0.0549 |
| **5** | Nep0.1 | 0.2775 | Keto | 0.0882 |  |  |


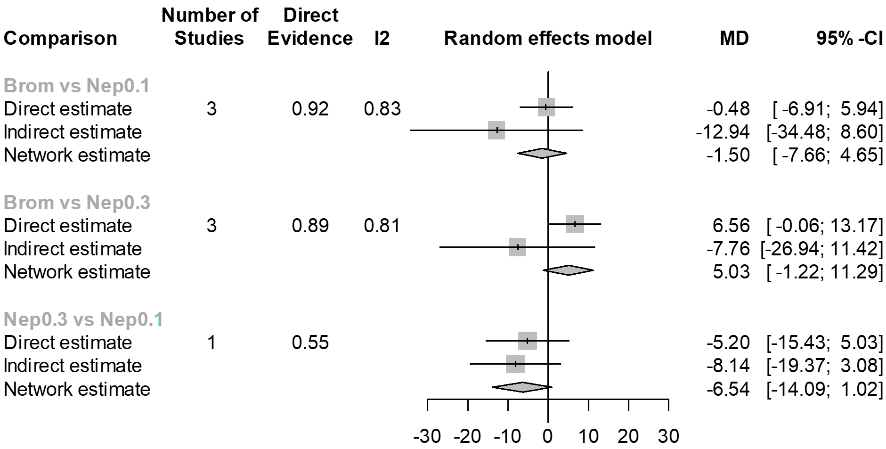


**Supplementary Figure S1 Net splitting analysis concerning foveal thickness.**

I2, inconsistency index; MD, mean difference; CI, confidence interval; Brom, Bromfenac; Keto, Ketorolac; Nep0.1, Nepafenac 0.1%; Nep0.3, Nepafenac 0.3%.


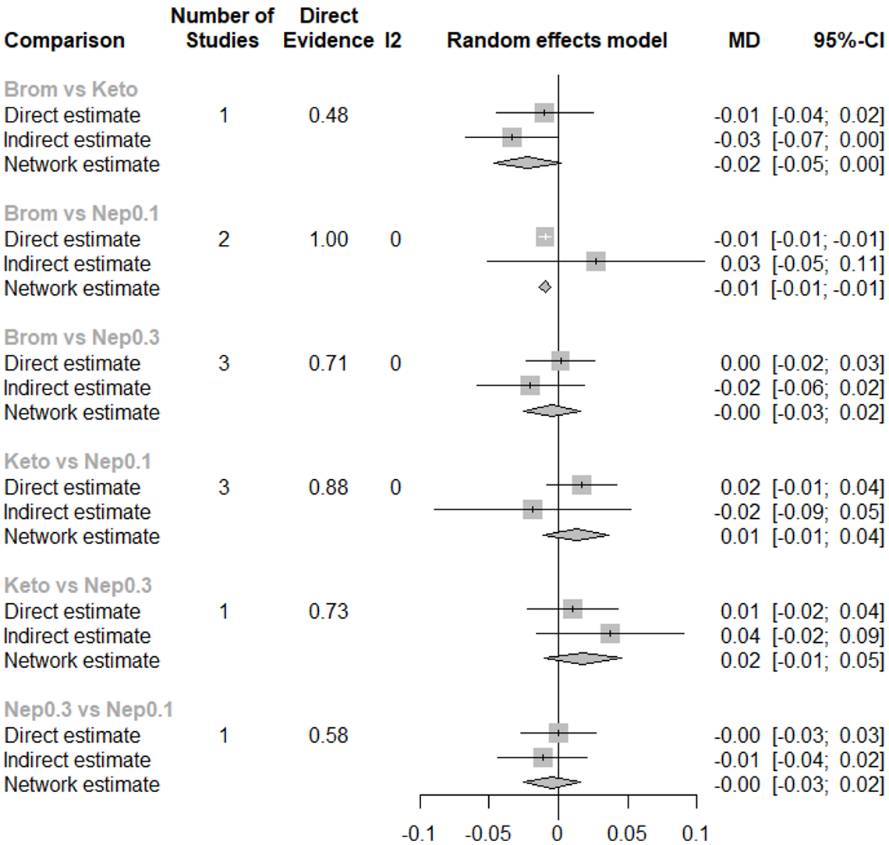


**Supplementary Figure S2 Net splitting analysis concerning BCVA.**

I2, inconsistency index; MD, mean difference; CI, confidence interval; Brom, Bromfenac; Keto, Ketorolac; Nep0.1, Nepafenac 0.1%; Nep0.3, Nepafenac 0.3%.


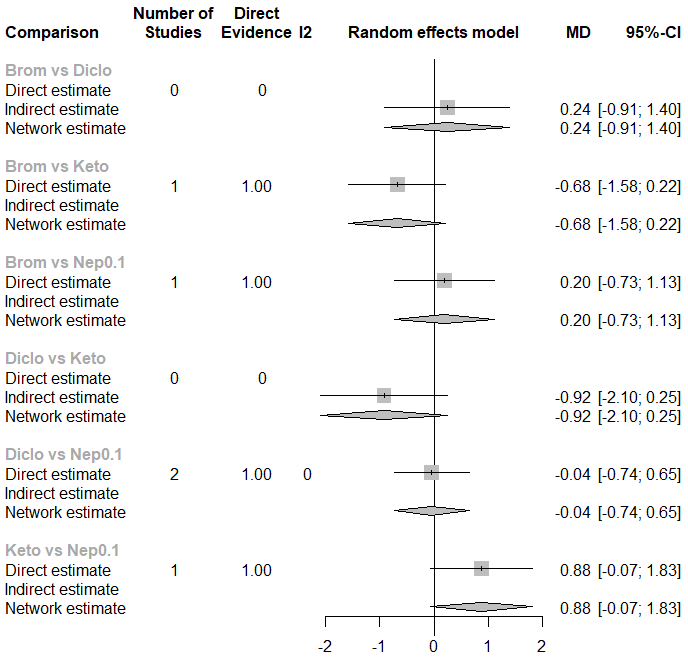


**Supplementary Figure S3 Net splitting analysis concerning IOP.**

I2, inconsistency index; MD, mean difference; CI, confidence interval; Brom, Bromfenac; Diclo, Diclofenac; Keto, Ketorolac; Nep0.1, Nepafenac 0.1%; Nep0.3, Nepafenac 0.3%.

Due to low heterogeneity, there was no need for a sensitivity analysis.


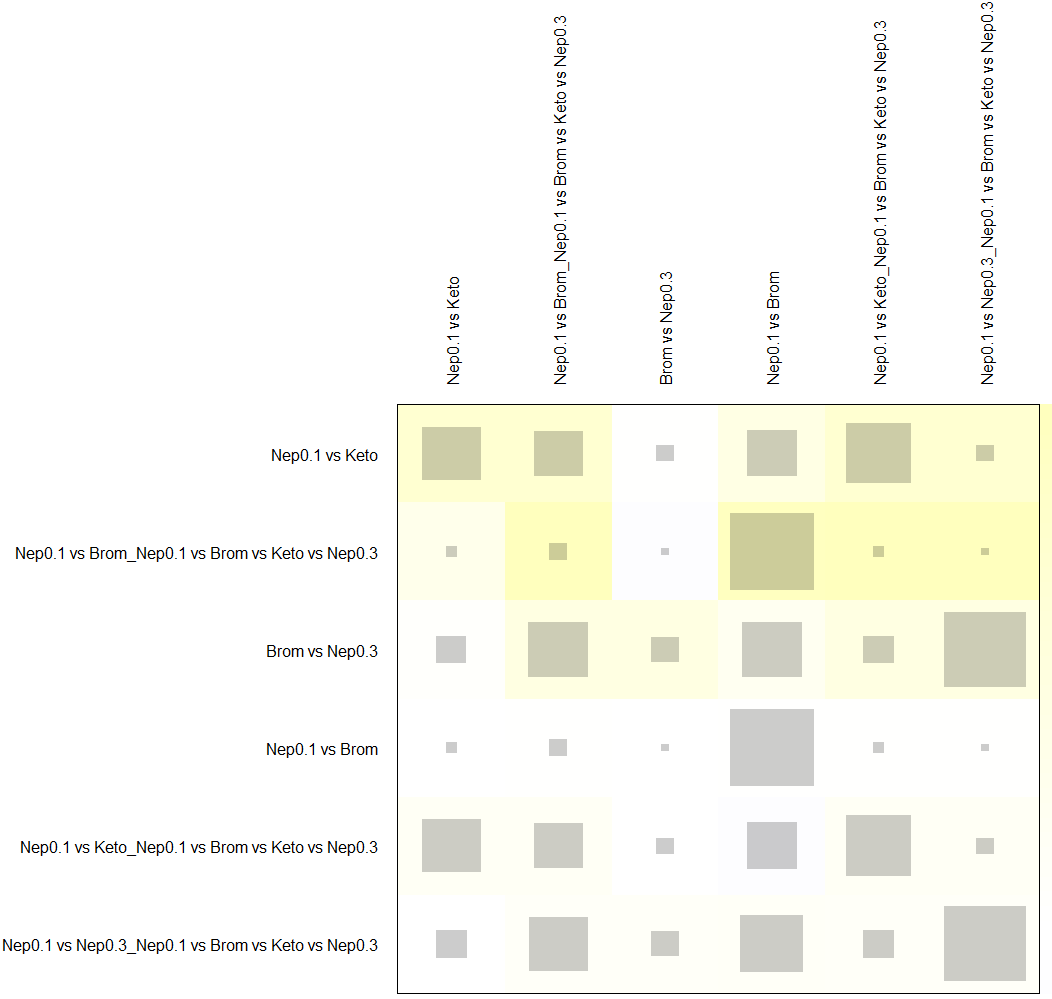


**Supplementary Figure S4. Net heat plot concerning BCVA.**





**Supplementary Figure S5. Individual study quality assessment**
